# Rapid-onset DRESS syndrome secondary to iopamidol contrast media with subsequent syndrome of inappropriate antidiuretic hormone secretion

**DOI:** 10.1016/j.jdcr.2024.04.008

**Published:** 2024-04-18

**Authors:** Rylee Moody, Daniel Tinker, M. Yadira Hurley, Gillian Heinecke

**Affiliations:** a Saint Louis University, School of Medicine, St. Louis, Missouri; b Department of Dermatology, SSM Health SLUCare, St. Louis, Missouri

**Keywords:** contrast, drug reaction with eosinophilia and systemic symptoms, HHV-6, iopamidol contrast, J-SCAR, RegiSCAR, syndrome of inappropriate anti-diuretic hormone

## Introduction

Drug reaction with eosinophilia and systemic symptoms (DRESS) syndrome is a severe delayed hypersensitivity reaction characterized by rash, fever, hematologic abnormalities, lymphadenopathy, and organ involvement.^1^^,^^2^ Prompt recognition is crucial as DRESS can be life-threatening.^3^ The typical latency period between exposure to the offending drug and onset is 2 to 8 weeks and is a diagnostic parameter in DRESS scoring systems.^3^ Few reports of DRESS induced by iodinated contrast exist; however, amongst the described cases, symptoms began within 48 hours.^1^^,^^4^ We report a similar case of DRESS induced by iopamidol contrast who went on to develop syndrome of inappropriate antidiuretic hormone release (SIADH).

## Patient presentation

**Fig 1:**
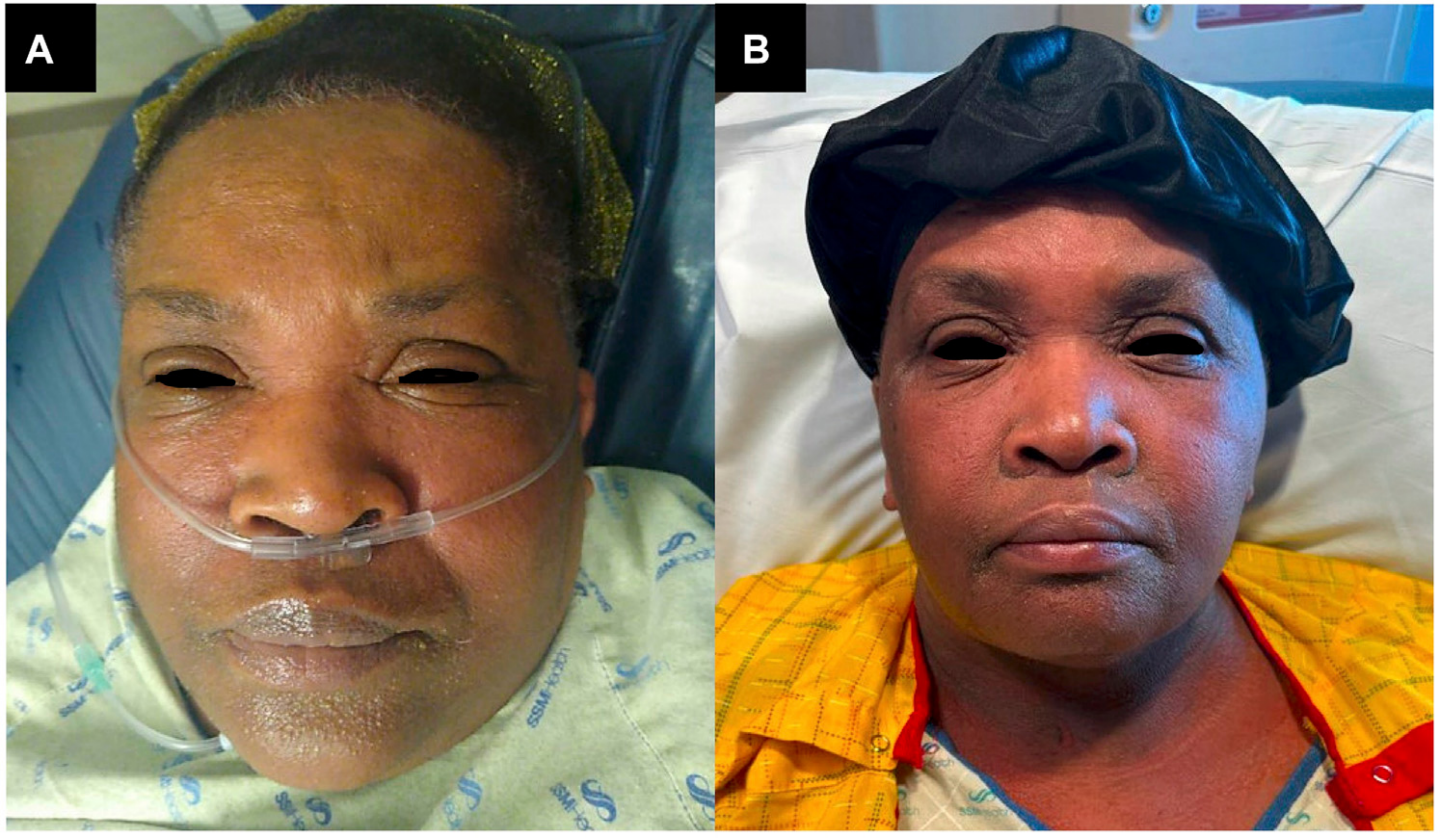
**A,** Photo of facial swelling and erythema at initial presentation. **B,** Photo of facial erythema following additional exposure to iopamidol contrast.

A 65-year-old African American female with history of peripheral artery disease, hypertension, and cerebral aneurysms presented with facial swelling, a pruritic rash on the face, trunk, and upper arms, generalized weakness, shortness of breath, and fever (105 °F) (Fig 1). These symptoms began 2 days after receiving computed tomography (CT) angiography with iodinated contrast of the lower extremities. No new medications had been ingested in the prior 12 weeks. She reported a prior history of rash with iodinated contrast several years ago.

She was treated with subcutaneous epinephrine, intravenous Benadryl, and methylprednisolone for suspected anaphylaxis. After 72 hours of persistent facial swelling, computed tomography (CT) angiography of the head/neck was performed due to concern for aneurysmal rupture or superior vena cava syndrome. Imaging was negative; however, her condition progressed. Laboratory workup revealed eosinophilia of 1900/microliter, atypical lymphocytes, elevated troponins and elevated transaminases with alanine transaminase of 142 and aspartate aminotransferase of 100.

**Fig 2:**
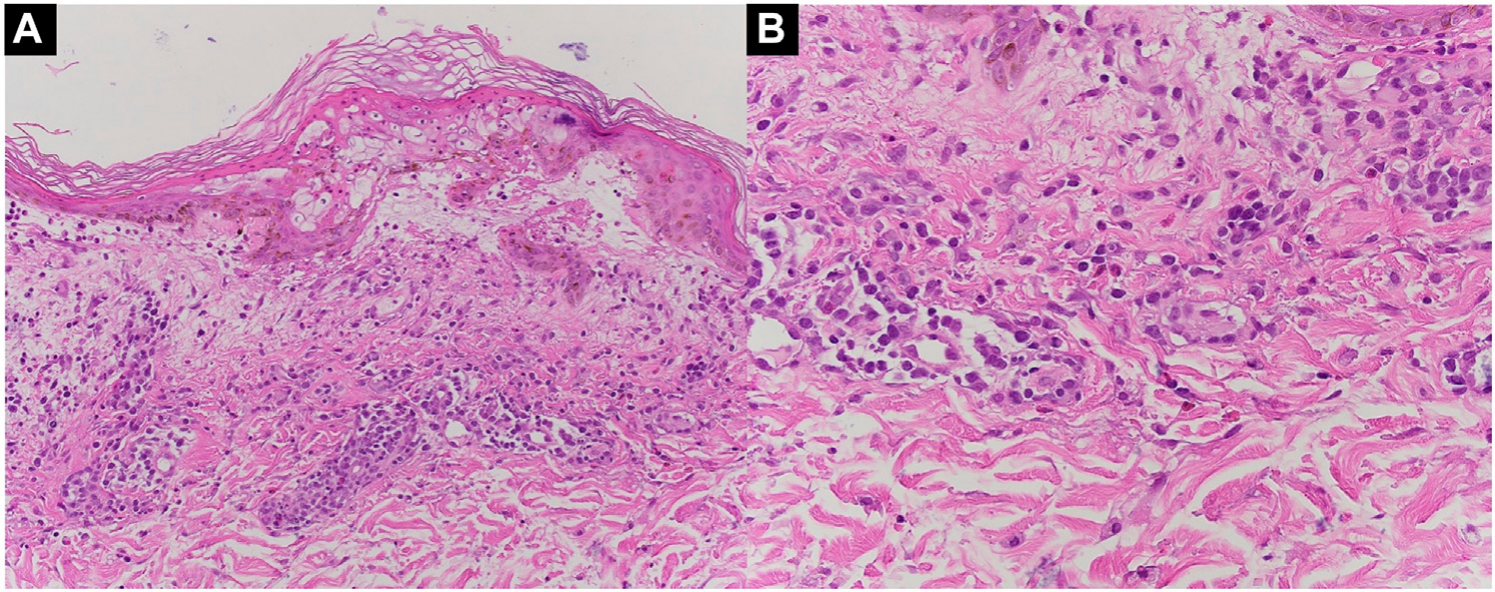
There is epidermal necrosis. The dermis shows marked papillary edema with a brisk inflammation composed of lymphocytes, neutrophils, and eosinophils. (Hematoxylin-eosin stain; original magnifications: (**A**) ×200; (**B**) ×400)

Biopsies from duskier plaques on her left and right arm revealed epidermal necrosis, marked dermal edema, and brisk inflammation composed of eosinophils, lymphocytes, and neutrophils (Fig 2). There was some perifollicular involvement of the mixed inflammatory infiltrate. No pustules were noted clinically or histologically.

This, in combination with her rash, facial and lower extremity edema, infiltration, scaling, cervical lymphadenopathy, and 2 sites of organ involvement (liver, heart) resulted in a RegiSCAR score of 6, making the diagnosis of DRESS “definite”. She was restarted on IV methylprednisolone 1 mg/kg and DRESS reaction to iodinated contrast was recorded.

One month after discharge, still on prednisone, she presented with fatigue and generalized weakness. Her sodium had acutely dropped to 124 mmol/L from her prior levels in the mid 140’s mmol/L. She was diagnosed with SIADH. Diuretics were held, and she was placed on fluid restriction, which normalized her sodium. HHV-6 test was ordered but not performed due to laboratory error.

## Discussion

DRESS, a severe form of drug hypersensitivity, has potential for significant morbidity and a mortality risk of 10%.^2^^,^^5^ Common culprits include anticonvulsants, lamotrigine, allopurinol, sulfonamides, and antibiotics.^1^ Multiple scoring systems have been established, including the Japanese Severe Cutaneous Adverse Reaction (J-SCAR) and the Registry of Severe Cutaneous Adverse Reactions (RegiSCAR) validation scoring system.^2^ A study comparing J-SCAR and RegiSCAR criteria found that J-SCAR failed to diagnose a significant proportion of DRESS cases, even in severe cases.^2^^,^^6^ J-SCAR’s poorer sensitivity may be because it requires the rash onset to be at least 3 weeks after initiation of the provoking drug to qualify for DRESS diagnosis. Thus, RegiSCAR is the most detailed and frequently used to diagnose DRESS.^2^ Iodiated contrast in addition to antibiotics can cause DRESS onset in less than 15 days.^1^^,^^4^

SIADH has previously been reported in a patient with DRESS with HHV-6 reactivation and limbic encephalitis.^7^ HHV-6 induced-limbic encephalitis can cause SIADH, and is most commonly seen following hematopoietic cell transplants.^8^ In a patient with recent history of DRESS with SIADH non-responsive to first-line treatment, empiric treatment with valganciclovir to treat HHV-6, a virus that over 90% of people have been exposed to, should be considered.^9^

We report a case of exceedingly rapid onset DRESS due to iopamidol contrast, administered twice in a 72-hour period, likely worsening the severity of the disease. She subsequently developed SIADH. This case emphasizes the importance of being aware of the variability in DRESS presentations and the significance of early recognition, given DRESS’ life-threatening nature and potential for significant morbidity.

## Conflicts of interest

None disclosed.
